# A Secure High-Order Gene Interaction Detecting Method for Infectious Diseases

**DOI:** 10.1155/2022/4471736

**Published:** 2022-04-21

**Authors:** Huanhuan Wang, Hongsheng Yin, Xiang Wu

**Affiliations:** ^1^School of Information and Control Engineering, China University of Mining and Technology, Xuzhou221116, China; ^2^School of Medical Information & Engineering, Xuzhou Medical University, Xuzhou 221000, China

## Abstract

Infectious diseases pose a serious threat to human life, the Genome Wide Association Studies (GWAS) can analyze susceptibility genes of infectious diseases from the genetic level and carry out targeted prevention and treatment. The susceptibility genes for infectious diseases often act in combination with multiple susceptibility sites; therefore, high-order epistasis detection has become an important means. However, due to intensive computational burden and diversity of disease models, existing methods have drawbacks on low detection power, high computation cost, and preference for some types of disease models. Furthermore, these methods are exposed to repeated query and model inversion attacks in the process of iterative optimization, which may disclose Single Nucleotide Polymorphism (SNP) information associated with individual privacy. Therefore, in order to solve these problems, this paper proposed a safe harmony search algorithm for high-order gene interaction detection, termed as HS-DP. Firstly, the linear weighting method was used to integrate 5 objective functions to screen out high-order SNP sets with high correlation, including K2-Score, JS divergence, logistic regression, mutual information, and Gini. Then, based on the Differential Privacy (DP) theory, the function disturbance mechanism was introduced to protect the security of individual privacy information associated with the objective function, and we proved the rationality of the disturbance mechanism theoretically. Finally, the practicability and superiority of the algorithm were verified by experiments. Experimental results showed that the algorithm proposed in this paper could improve the detection accuracy to the greatest extent while guaranteeing privacy.

## 1. Introduction

The prevention and treatment of infectious diseases is an important and long-term task for human beings. The Genome Wide Association Studies (GWAS) can analyze susceptibility genes from the whole gene range and carry out targeted prevention and treatment of infectious diseases, which is of great significance for the long-term development of human beings. More and more studies showed that the interaction between genes is the main cause of genetic variation in infectious diseases [1]. Detection of gene interaction refers to the search for multiorder gene site combinations affecting diseases to determine the pathogenic cause and genetic mechanism, which has become an important research direction in Genome Wide Association Studies (GWAS) [2, 3].

Thousands of methods for identifying gene interactions have been studied, and they can be divided into exhaustive [4], random [5], filtering [6], modeling [7], and intelligent methods [8]. The exhaustive method identifies genes that interact by combining all the possibilities. The random method extracts only partial gene combinations from the data to analyze the disease model. The former method has comprehensiveness and completeness, but the calculation burden is too heavy, and the detection accuracy of the latter still needs to be improved. The filtering method is a combination of exhaustive and filtering method, while reducing the computational burden, still misses some important combinations of gene interactions. Modeling is based on traditional machine learning methods, which use probabilistic methods to identify gene interactions, but this method still cannot detect gene combinations with marginal effects.

The latest breakthrough for identifying gene interactions in GWAS is swarm intelligence search method, which makes full use of the information contained in current optimization parameters to generate final new search results, and has become one of the most popular methods, for example, AntEpiSeeker [9], IEACO [10], FHSA-SED [11], IOBLPSO [12], DECMDR [13], and other methods.

**Figure 1:**
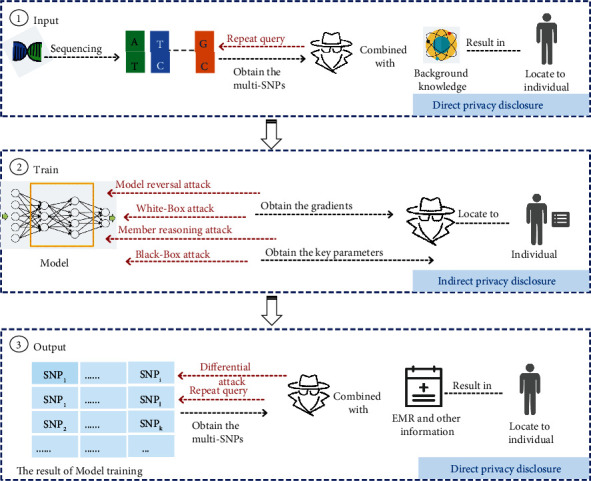
The privacy disclosure of high-order gene interaction detection.

Although swarm intelligence search method has the advantages of controllable time complexity compared with other methods, it still faces many serious problems. First, the evaluation function design of swarm intelligence method is unreasonable [14]. The evaluation function is not selected from multiple dimensions, so the auxiliary optimization search strategy has defects. Second, the accuracy of identifying the results of gene interactions still needs to be improved [15]. Last but not least, there are privacy risks associated with identifying multiorder genetic interactions [16, 17]. As shown in Figure 1, there are risks of privacy disclosure in the input, training, and output stages of genetic data. In the data input stage, the untrusted third party launches repeated query attack to obtain the original data information for many times and locate the individuals by combining these information with the background knowledge. In the model training stage, the attacker can obtain the gradient and some key parameters directly related to the original data through various means such as model inversion, so as to mine more personal information based on the background knowledge. This process is an indirect privacy breach. In the data output stage, the untrusted third party obtains more genetic detection result information by differential privacy budget attack. Combining this information with a genetic history of medical visits for certain diseases can target individuals. Therefore, the privacy security of genetic data needs to be solved urgently.

Therefore, in order to solve the above problems, this paper proposed a safe Harmony Search (HS) algorithm for high-order gene interaction detection. Firstly, the linear weighting method was used to integrate various objective functions, including K2-Score, JS divergence, logistic regression, mutual information, and Gini, to screen out the Single Nucleotide Polymorphism (SNP) solution set with high correlation. Then, to protect the privacy security of sensitive information, we introduced the function disturbance mechanism and analyzed the rationality of this mechanism. Specific contributions are as follows:

- Select fitness functions from different dimensions to overcome the difficulty of poor detection results caused by gene interaction among functions in the same gradient. Use the linear weighted sum method to combine these functions in the same gradient direction for comprehensive evaluation of the identified multiorder gene combinations
- Propose a privacy protection mechanism to solve the problem of privacy disclosure in high-order gene interaction identification. Specifically, this mechanism interferes with the polynomial coefficients of the objective optimization function rather than simply adding noise to the result, achieving the balance between privacy and utility
- Theoretical and experimental results show that HS-DP can not only identify the accuracy of gene interaction but also protect the security of SNP information associated with individual privacy

The remainder of the paper is organized as follows.  Section 2 overviews the related works. List the preliminaries of this paper in Section 3. In Section 4, we introduce our algorithm in detail. The experimental evaluations and results are discussed in Section 5. Finally, Section 6 summarizes the paper.

## 2. Related Work

Gene interaction refers to the influence of the interaction between two or more single nucleotide polymorphisms (or genes) on the phenotype. Epistasis is one of the important genetic factors affecting complex diseases. The swarm intelligence search algorithm is widely used in the interactive recognition of SNPs because it can efficiently and quickly search for feasible solutions within a given range. Commonly used swarm intelligence search algorithms include particle swarm optimization algorithm, differential evolution algorithm, artificial bee colony algorithm, and ant colony optimization algorithm.

In the study of Particle Swarm Optimization (PSO), Yang et al. [18] proposed an Odds Ratio-Based binary Particle Swarm Optimization (OR-BPSO) method to evaluate the risk of breast cancer. BPSO provides the combinational SNPs with their corresponding genotype, called SNP barcodes, with the maximal difference of occurrence between the control and breast cancer groups. Chuang et al. [19] proposed IPSO algorithm to improve the reliability of PSO for the identification of the best protective SNP barcodes associated with breast cancer. The top five SNP barcode results are retained for computing the next SNP barcode with a one-SNP increase for each processing step. Shang et al. [12] proposed an improved opposition-based learning particle swarm optimization for the detection of SNP-SNP interactions. The proposed algorithm enhances the global exploration capability and also avoids premature convergence. The particles cover a wider search space and perform in-depth search on highly suspicious SNP sets. Chuang et al. [20] applied an evolutionary algorithm to facilitate statistical methods in the analysis of associated variations for disease susceptibility. The Gauss particle swarm optimization algorithm was used to detect and identify the best protective association (i.e., combinations of SNPs with genotypes) with breast cancer. Yang et al. [21] proposed a PSO-Based Multifactor Dimensionality Reduction approach (PBMDR). MDR was used to detect multilocus interactions based on the PSO algorithm. Chuang et al. [22] proposed to combine chaotic graphs with PSO methods to detect the interaction of SNPs in high-dimensional datasets, where chaotic graphs help the PSO algorithm to avoid falling into local optima.

In the study of Differential Evolution (DE). Yang et al. [13] proposed a new algorithm which combines the DE algorithm with a Classification-based Multifactor Dimensionality Reduction (CMDR), termed DECMDR. Yang et al. [23] proposed a catfish Taguchi-based binary differential evolution (CT-BDE) algorithm for identifying SNP-SNP interactions. In the search space, the catfish effect prevents the premature convergence of the population, and the Taguchi method improves the search ability of the BDE algorithm. Guan et al. [24] proposed a fast evolutionary optimization method named search-history-guided differential evolution. This method applies the search history memorized in a binary space partitioning tree to enhance its power for selecting feature combinations. Guan et al. [25] proposed a two-stage algorithm DEseeker to detect epistatic effects. This scheme can identify hidden SNPs, but it takes too much time to execute in large-scale datasets.

In the study of Artificial Bee Colony (ABC). Yang et al. [26] proposed a method of superiority mining based on the artificial bee colony algorithm to optimize the Bayesian network. The algorithm is applied to the Bayesian network heuristic search strategy. Li et al. [27] proposed and formulated a decomposition-based upper interactive multiobjective artificial bee colony algorithm. Two objective functions are formulated to characterize various upper models and a rank probability model based on the fast nondominated ranking method is proposed. After that, a local search algorithm based on mutual information was proposed.

In the study of Ant Colony Optimization (ACO). Wang et al. [9] proposed a new tool for discovering apparent interactions in large-scale case control studies, which uses a two-stage optimization program. Moreover, Wang et al. [28] developed AntEpiSeeker2.0. By looking at pheromone distribution across pathways, epistasis-associated pathways can be easily identified. Sinnott-Armstrong et al. [29] implemented ACO MDR on the GPU. The performance advantages of GPUs combined with the computational efficiency of heuristic evolutionary algorithms can solve larger-scale problems. Li et al. [30] proposed a novel approach which could find a gene-gene interaction model consists of a flexible number of susceptible loci based on ACO strategy. The proposed method becomes a potential solution for finding the complex association rules between susceptible SNP subsets and common human diseases in the future. Shang et al. [31] introduced an algorithm based on ACO, which by incorporating heuristic information into ant decision rules. Introduce heuristic information in the search process, and perform a chi-square test during the iteration. When the iterative process is completed, sort and use postprocedures to filter. Jing and Shen [32] proposed a multiobjective ACO algorithm to detect genetic interactions, which combines the standard logistic regression and Bayesian network methods, and also design a memory-based multiobjective ACO algorithm. Liu et al. [33] proposed a flexible two-stage method (called HiSeeker) to detect high-level interactions. In the screening phase, HiSeeker uses chi-square test and logistic regression model. In the search phase, exhaustive search and search based on ant colony optimization are used. HiSeeker can detect high-level interactions more efficiently and effectively. Sapin et al. [34] introduced an ACO-based algorithm called to identify all possible binding sites of transcription factors from upstream of coexpressed genes, this algorithm uses the powerful optimization capabilities of ACO, which can not only improve the accuracy of the results but also achieve very high speeds. Sun et al. [35] proposed an algorithm based on ACO and a new fitness function value, which combines Bayesian networks and mutual information to detect SNP-SNP interactions. Guan et al. [36] proposed a new ACO algorithm based on automatic adjustment mechanism to solve the problem of combinatorial explosion of stratum by mining apparent interaction from large-scale data. The mechanism automatically adjusts the behavior of artificial ants based on real-time feedback information, so that the algorithm can run to the best state. Guan improved and proposed SEPACO [37], a self-evolved parameter based on ACO algorithm.

## 3. Preliminaries and Backgrounds

In this section, we will introduce the related concepts and background knowledge of the HS algorithm and the Differential Privacy (DP) mechanism.

### 3.1. HS Algorithm

The HS algorithm is inspired by the music-making process of jazz musicians, who improvise the pitches of their instruments in search of perfect harmony [38]. It is a group-based metaheuristic algorithm, whose idea is to realize the cognition of unknown complex problems through information exchange and learning among individuals in a group. More precisely, harmony and its tonal set are analogous to the candidate solution and its decision variable set *X* = (*x*_1_, *x*_2_, ⋯*x*_*N*_), respectively. In addition, the measurement of harmony's pleasurable state by the audience's aesthetic evaluation corresponds to an objective function *f*(·). Each attempt by a musician to progressively improve harmony by producing some new pitch corresponds to the application of search operators that change the value of some decision variable during each iteration of the harmony search. The musician's memory is where good harmony is stored, similar to the solution, called *HM* in the harmony algorithm.

(1) $$\begin{array}{c} HM=\left[\begin{array}{c} X^{1}\\ X^{2}\\ \cdots \\ X^{HMS} \end{array}\left|\begin{array}{c} f\left(X^{1}\right)\\ f\left(X^{2}\right)\\ \cdots \\ f\left(X^{HMS}\right) \end{array}\right.\right]=\left[\begin{array}{cccc} x_{1}^{1} & x_{2}^{1} & \cdots & x_{N}^{1}\\ x_{1}^{2} & x_{2}^{2} & \cdots & x_{N}^{2}\\ \cdots & \cdots & \cdots & \cdots \\ x_{1}^{HMS} & x_{2}^{HMS} & \cdots & x_{N}^{HMS} \end{array}\left|\begin{array}{c} f\left(X^{1}\right)\\ f\left(X^{2}\right)\\ \cdots \\ f\left(X^{HMS}\right) \end{array}\right.\right]. \end{array}$$

HS algorithm has been used in gene interaction recognition. Tuo's team pioneered the FHSA-SED approach [11], which is an integrated HS and local search approach for identifying 2-order gene interaction recognition studies. In this method, K2 score and Gini score were selected to represent SNP loci, and local search algorithm with two-dimensional Tabu table was used to screen some disease models with strong epistatic effects. Although FHSA-SED has higher detection accuracy than other intelligent algorithms, its rationality still needs to be verified. In 2017, this team proposed validation in a AMD, to demonstrate the rationality and research ability of HS algorithms for identifying gene interactions [39]. However, the experimental results show that the accuracy of this method still needs to be improved. Recently, the team expanded the research content and proposed MP-HS-DHSI algorithm [40]. A new evaluation standard of harmony target is designed and G-test is integrated as verification method. In general, this method greatly improves the detection accuracy but ignores the data security problem, and the comprehensiveness of the objective evaluation function still needs to be considered. On this basis, we propose a safe HS algorithm for high-order gene interaction detection.

### 3.2. Differential Privacy

DP is a set of mechanisms developed for data analysis on sensitive data. By obfuscating database query results, the privacy of data at the personal level is realized and the query results are approximately correct. Before we introduce the definition of differential privacy, let us first agree on some symbols.

Define dataset as the *D*, each row of the data takes a value in this set. The number of rows in our fixed database is *n*, then, a database *x* is an element in the power set *D*^*n*^ (the set composed of all subsets of *D*. The query is defined as a function *q*, enters the database *x*, and outputs certain values. *M*(*x*) is the random mechanism attached to the query *q*, Enter a database *x* to get a randomized query result. *x* is the database random variable, *X*_*i*_ represents the content of the *i*-th row of the database. *p* is the mapping defined on the database row. Pr is the probability distribution.

**Definition 1 . (ε-Differential Privacy) [41].**

If for each pair of databases *x* and *x*′ with only one row that are not the same, and the output *y* of each possible *M*(*x*), all satisfy

(2) $$\begin{array}{c} \mathrm{Pr}\left[M\left(x\right)=y\right]\leq e^{\varepsilon }\mathrm{Pr}\left[M\left(X^{\prime }\right)=y\right], \end{array}$$

where *ε* > 0, and *δ* represents the event that the ratio of the probabilities for two adjacent datasets *x*, *y* cannot be bounded by *e*^*ε*^  after adding a privacy preserving mechanism. With an arbitrarily given *δ*, a privacy preserving mechanism with a larger *ε* gives a clearer distinguish ability of neighboring datasets and hence a higher risk of privacy violation.

After introducing the definition of difference, we will introduce three different noising mechanisms for differential privacy.

#### 3.2.1. Laplace Mechanism

The Laplace mechanism is a mechanism that satisfies differential privacy and is mainly used in counting queries. And, the query returns a vector of nonnegative integers, and only the case where the query returns a nonnegative integer is considered here. We now define a quantity called *l*_1_ sensitivity.

**Definition 2 . (Sensitivity) [42].**

The *l*_1_  sensitivity of a function *f* : *D*^*n*^⟶*R*^*k*^.

(3) $$\begin{array}{c} \Delta f=\max_{\begin{array}{c} x,y\in D^{n}\\ {\left\|x-y\right\|}_{1}=1 \end{array}}{\left\|f\left(x\right)-f\left(y\right)\right\|}_{1,}. \end{array}$$

where Δ*f* means single record changes the output of *f*, how much can be changed at most.  Δ*f* can be used to control the amplitude of noise.

Define the Laplace mechanism for any function *f* : *D*^*n*^⟶*R*^*k*^ as

(4) $$\begin{array}{c} M_{L}\left(x,f\left(\cdot \right),\varepsilon \right)=f\left(x\right)+{\left(Y_{1},\cdots ,Y\right)}_{k}, \end{array}$$

where *Y*_*i*_ is an independent and identically distributed (iid) random variable sampled from *Y*_*i*_ ~ *Lap*(Δ*f*/*ε*).

#### 3.2.2. Gaussian Mechanism

The Gaussian mechanism mainly provides differential privacy protection for numerical data. The Laplace mechanism provides a strict (*ε*, 0) − *DP*, while the Gaussian mechanism provides a relaxed (*ε*, *δ*) − *DP* mechanism.

For any $\delta \in \left(0,1\right),\sigma >\sqrt{2\ln\left(1.25/\delta \right)}\Delta f/\varepsilon$, noisy *Y* ~ *N*(0, *σ*^2^) satisfies (*ε*, *δ*) − *DP*.

(5) $$\begin{array}{c} P\left(M\left(x\right)\in S\right)\leq e^{\varepsilon }P\left(M\left(y\right)\in S\right)+\delta , \end{array}$$

where *M*(*x*) = *f*(*x*) + *Y*, there are three main parameters here. The standard deviation of the Gaussian distribution *σ*, which determines the scale of the noise. *ε*  indicates the privacy budget, which is negatively correlated with noise. *δ* represents the relaxation term. For example, if it is set to 10^−5^, it means that the probability of 10^−5^ can only be tolerated, which violates strict differential privacy.

#### 3.2.3. Exponential Mechanism

Exponential mechanism is used for differential privacy protection of nonnumerical data. The overall idea of the exponential mechanism is that when a query is received, it does not output a *O*_*i*_ result exactly but returns the result with a certain probability value, thereby achieving differential privacy. And this probability value is determined by the scoring function, the output probability of the high score is high, and the output probability of the low score is low.

**Definition 3 . (Utility function sensitivity).**

Utility function *u* : *D*^*n*^ × *O*⟶ℝ, mapping (database-query output) to utility score, we define the sensitivity of the utility function *u* as

(6) $$\begin{array}{c} \Delta u\equiv \max_{r\in R}\max_{x,y:{\left\|x-y\right\|}_{1}\leq 1}\left|u\left(x,o\right)-u\left(y,o\right)\right|. \end{array}$$

Exponential mechanism *M*_*E*_(*x*, *u*, *O*). The probability of selecting and outputting a result *o* ∈ *O* is proportional to exp(*εu*(*x*, *o*)/2Δ*u*). But exp(*εu*(*x*, *o*)/2Δ*u*) does not express the probability value, so it is necessary to normalize all possible values to get the corresponding probability value.

(7) $$\begin{array}{c} \mathrm{Pr}\left[O_{i}\right]=\frac{\exp\left(\varepsilon \left(D,O_{i}\right)/2\Delta u\right)}{\sum_{j}\exp\left(\varepsilon \left(D,O_{j}\right)/2\Delta u\right)}. \end{array}$$

Finally, choose an output with a higher utility score with a higher probability.

## 4. Our Proposed HS-DP Scheme

**Figure 2:**
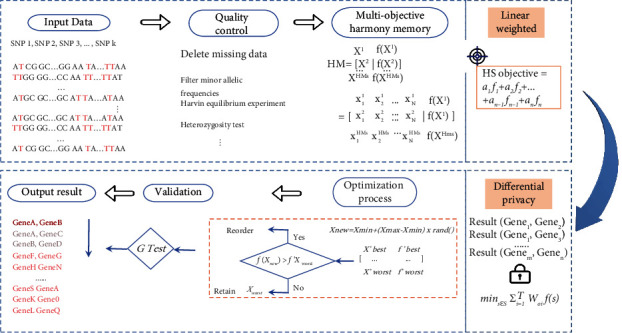
HS-DP overview.

Current studies on gene interactions are still focused on identifying 2-order gene interactions, but lack of higher-order gene interactions. In addition, few researchers have focused on the security of gene interaction GWAS based. In order to solve the current problems, a framework for high-order gene interaction detection HS-DP based on secure harmony search is proposed in this paper. This framework provides privacy guarantee for high-order gene interactions, not only effectively preserves privacy information in training data but also ensures the availability of the framework through adaptive functional perturbation mechanism. As shown in Figure 2, HS-DP mainly consists of 7 steps, including standardized data input, data quality control, multiobjective memory seize design, linear combination, differential perturbation, verification, and outputting results. Some of the key steps are detailed below. In addition, the definition of high-order gene interaction and specific problems will also be introduced in the following.

### 4.1. High-Order Gene Interaction

Studies have shown that almost no phenotypic characteristics of an individual are determined by a single gene, so gene-gene (or gene-environment interaction) has important theoretical and practical significance in explaining individual characteristics. High-order gene interaction is defined as the combinations of at least *K* SNPs affecting phenotype or disease genes. We expressed the gene interactions process as *R* = {*S*, *G*, *A*}, where *S* = {*S*_1_, *S*_2_ ⋯ , *S*_*i*_} represented SNP typing, *G* = {*G*_11_, *G*_12_, ⋯, *G*_*ij*_} represented interaction between *G*_*i*_ and *G*_*j*_ corresponding genes, and *A* = {*A*_1_, *A*_2_ ⋯ , *A*_*i*_} represented association results. The *K*-order gene interaction represents the recognition of SNP interaction results of the order of 3^*n*^. Among them, when *G*_*mn*_ > *θ*, *G*_*mn*_ is called the result with the main effect, and when *G*_*ab*_ < *θ*, *G*_*ab*_ is the result of the edge effect.

### 4.2. Problem Statement

Let the set of gene variables *X* = {*X*_1_, *X*_2_, ⋯, *X*_*i*_} includes *S* = {*S*_1_, *S*_2_, ⋯, *S*_*j*_} SNP marker for *N* individuals. For high-order gene interaction detection algorithms, the temporal *O*(*f*(*n*)) and spatial *S*(*n*) complexity of the algorithm increases exponentially in 3^*n*^ detection demand. There are three ways in which neural network training data may reveal genetic privacy. In the data input phase, one attacker *A* initiates *AK* = {*AK*_1_, *AK*_2_, ⋯, *AK*_*n*_} attacks, including repeated query, to obtain the original SNP data information *I*_1_, *I*_2_, ⋯*I*_*n*_ for several times and locate individuals based on the background information *KN* = {*KN*_1_, *KN*_2_, ⋯, *KN*_*n*_}. In the model training stage, *A* can obtain gradients and some key parameters {*α*, *θ*, ⋯, *μ*} directly related to the original data through *AK* = {*AK*_1_, *AK*_2_, ⋯, *AK*_*n*_} such as model inversion, so as to mine more personal information based on *KN* = {*KN*_1_, *KN*_2_, ⋯, *KN*_*n*_}. In the data output stage, *A* obtains more genetic detecting results information through *AK* = {*AK*_1_, *AK*_2_, ⋯, *AK*_*n*_} such as differential privacy budget. Combining this information with the genetic history of visits for certain diseases can identify individuals.

#### 4.2.1. K2-Score

Initially, the *Bayesian network* (*BN*) is a graphical statistical model that represents the dependence of some random variables, and its reasoning model by directed acyclic graph, where nodes denote random variables and edges denote dependence between two link nodes. *BN* is also used to identify gene interaction among SNP, which calculates the association of variants and genetic genotype. In general, there are directed links from SNP *M*_*i*_ to genotype status *T* when *M*_*i*_ associates with *T*. From more than 20 kinds of *BN* models [40], HS-DP selects *K2-score* to assess the effects of SNP combinations and genotype. More details about *K2-score* based on *Bayesian Network* are introduced in literature [40]. The *K2-score* function can be written as Equation (8). In a word, lower *K2-score* value shows more stronger association between SNP combinations and the genetic phenotype.

(8) $$\begin{array}{c} k2-Score=\overset{N}{\underset{n=1}{\prod }}\left(\frac{\left(I-1\right)!}{\left(M_{i}+I-1\right)!}\overset{i}{\underset{i=1}{\prod }}M_{\text{in}}!\right). \end{array}$$

#### 4.2.2. JS Divergence

JS divergence, derived from Kullback-Leibler (KL) divergence [43], refers to the metric of symmetry between two probability distributions.

In GWAS, JS divergence can be used to measure SNP genotype deviation between case data and control data. For a SNP combination, the genotype distribution of case and control was set as *ρ*_case_ and *ρ*_control_, respectively. JS divergence between *ρ*_case_ and *ρ*_control_ can be expressed by Equation (9).

(9) $$\begin{array}{c} JS=0.5\left(\overset{I}{\underset{i=1}{\sum }}\overset{2}{\underset{j=1}{\sum }}\frac{n_{ij}}{n_{i}}\log\frac{2n_{ij}}{ni}\right), \end{array}$$

where *ρ*_case_ and *ρ*_control_ represent the ratio of the *i*-th genotype combination in the case and control samples, respectively. In general, when looking for gene interaction, the larger the JS divergence value is, the greater the difference between the genotype of the case group and the control group is, and the stronger the association between SNP combination and disease status is, that is, the gene pair has epistatic effect.

#### 4.2.3. Logistic Regression

Logistic regression method is often used to identify the interactions with SNPs with strong epistatic effects [44]. Let *M*_1_ and *M*_2_ denote two SNPs and *Y* is the result of gene interactions. In two-order SNP *M*_*i*_ and *M*_*j*_, HS-DP adopts a logistic regression model to identify the association between (*M*_*i*_ and *M*_*j*_) and disease status *D* (1 for yes and 0 for no) as follows:

(10) $$\begin{array}{c} \log\left(\frac{P\left(D=1\;|\;\left(M_{i},M_{j}\right)\right)}{P\left(D=0\;|\;\left(M_{i},M_{j}\right)\right)}\right)=\alpha_{0}+\alpha_{i}M_{i}+\alpha_{j}M_{j}+{dM}_{i}M_{j}, \end{array}$$

where *α*_*i*_ and *α*_*j*_ are the main effects for SNP *M*_*i*_ and *M*_*j*_, respectively. Using the Newton-Raphson method to search the optimization value $\hat{L}F$ of the maximum likelihood of Equation (10) iteratively.

#### 4.2.4. Mutual Information

HS-DP fits mutual information to identify which combinations are true gene interactions. Mutual information has become one of the widely used functions for measuring the correlation of two variables [45]; thus, the formula can be written as

(11) $$\begin{array}{c} MI\left(M;T\right)=H\left(M\right)+H\left(T\right)-H\left(M,T\right), \end{array}$$

in which *H*(*M*) is the entropy of *M*, *H*(*T*) is the entropy of *T*. *H*(*M*, *T*) is the joint entropy of *M* and *T*. *M* is the position of a variant that is a SNP combination, and *T* is the genetic phenotype.

The definition of entropy and the joint entropy can be written as

(12) $$\begin{array}{c} H\left(M\right)=-\overset{3}{\underset{i_{1}=1}{\sum }}\cdots \overset{3}{\underset{i_{n}=1}{\sum }}\left(p\left(M_{i_{1}},\cdots ,M_{i_{n}}\right)\cdot \log p\left(M_{i_{1}},\cdots ,M_{i_{n}}\right)\right), \end{array}$$

(13) $$\begin{array}{c} H\left(T\right)=-\overset{1}{\underset{i=0}{\sum }}\left(p\left(t_{i}\right)\cdot \log p\left(t_{i}\right)\right), \end{array}$$

(14) $$\begin{array}{c} H\left(M,T\right)=-\overset{3}{\underset{i_{1}=1}{\sum }}\cdots \overset{3}{\underset{i_{n}=1}{\sum }}\overset{1}{\underset{i=0}{\sum }}\left(p\left(M_{i_{1}},\cdots ,M_{i_{n}},t_{i}\right)\cdot \log p\left(M_{i_{1}},\cdots ,M_{i_{n}},t_{i}\right)\right), \end{array}$$

where *n* is the number of SNPs in SNP combinations, and *t* is the label of samples, and then, *p* represents the probability distribution function. In general, higher mutual information value shows more stronger association between SNP combinations and the genetic phenotype.

#### 4.2.5. Gini Score

The Gini index is a measure of dispersion that can be used to measure the impure nature of data partitions or the inequality between values of frequency distributions [46]. The correlation problem of gene interactions is essentially a dichotomous problem and can therefore be measured by the Gini coefficient. The Gini index is a diversity index, specifically defined as

(15) $$\begin{array}{c} \text{Gini}=\overset{I}{\underset{i=1}{\sum }}\rho_{i}\cdot \left(1-\overset{J}{\underset{j=1}{\sum }}\rho_{i,j}^{2}\right), \end{array}$$

where *ρ*_*i*,*j*_(*ρ*_*i*,*j*_ = *n*_*ij*_/*n*_*i*_) is the estimated probability that the *i*-th genotype combination is actually associated with phenotype *y*_*j*_. (1 − ∑_*j*=1_^*J*^*ρ*_*i*,*j*_^2^) represents the estimated probability of genotype combinations being misclassified as phenotypic *y*_*j*_. *ρ*_*i*_(*ρ*_*i*_ = *n*_*i*_/*L*) is the percentage of the *i*-th genotype combination in the sample set. The smaller the Gini coefficient is, the stronger the correlation between SNP combination and phenotype is, that is, the gene has epistasis.

### 4.3. Functional Differential Perturbation

HS-DP utilized the linear weighted sum method to perform the above multiple fitness assessment functions. Weight allocation is one of the most important steps in the composition process. In this study, formula (16) is used to assign the appropriate weight, and the calculation process is as follows:

(16) $$\begin{array}{c} \text{Power}=\frac{\text{True SNPs}}{\sum_{n=1}\text{S}\text{NPs}}, \end{array}$$

(17) $$\begin{array}{c} \min_{s\in S}\overset{T}{\underset{i=1}{\sum }}W_{o_{i}}f\left(s\right), \end{array}$$

(18) $$\begin{array}{c} W_{i}=\frac{{\text{Power}}_{i}}{\sum_{i=1}\text{P}\text{ower}},\overset{m}{\underset{i}{\sum }}W_{i}=1, \end{array}$$

where equation (17) is the objective optimization function of HS-DP; however, literature [47] had confirmed that the objective function directly related to the original data in optimization problems will leak data privacy information. The objective function of HS-DP is not only directly related to the original data, but also its results are directly related to privacy information. On this basis, this paper proposed a general differential privacy function perturbation framework for the study of high-order gene interactions in GWAS to solve the privacy leakage problem.

Before introducing the specific content of this method, we would first introduce the preliminaries. Let *O* be a set of *n* objectives {*O*_1_, *O*_2_, ⋯, *O*_*n*_} and *i* genes. For each goal *O* = (*O*_*i*1_, *O*_*i*2_, ⋯, *O*_*id*_, *f*_*i*_), we assume no loss of generality, *f*_*i*_ ≥ 0. Our goal is to build an *O*-based regression model (also known as the objective function *F*) that takes the predictions of *F* as inputs and outputs *S*_1_, *S*_2_, ⋯, *S*_*M*_. HS-DP shows that *F* is a linear regression model parameterized by *W*, and *W* is an *N*-dimensional vector, where the number of *j*-th (*j* ∈ {1, 2, ⋯, *n*}) is equal to the weight of *f*_*i*_ in *F*. To evaluate the accuracy of *W*, we define a cost function *W*^∗^ with *O*_*i*_ and *W* as inputs. According to the definition of linear regression cost function, the equation of parameter *W*^∗^ is as follows:

(19) $$\begin{array}{c} W^{*}=\arg\underset{W}{\min}\overset{i}{\underset{n=1}{\sum }}F\left(f_{i},W\right). \end{array}$$

**Definition 4 . (ϵ-differential privacy).**

The randomized algorithm *A* satisfies *ϵ*-differential privacy, if for any output *R* of *A* and for any two neighbor databases, we have

(20) $$\begin{array}{c} \mathrm{Pr}\left[A\left(D_{1}\right)=R\right]\leq e^{\int }\cdot \mathrm{Pr}\left[A\left(D_{2}\right)=R\right]. \end{array}$$

The regression task for genetic data returns the parameter *W*^∗^ that minimizes the objective optimization function *F* = min_*s*∈*S*_∑_*i*=1_^*T*^*W*_*o*_*i*__*f*(*s*). Releasing *W*^∗^ directly would compromise the privacy of information that reveals gene data. In general, our method perturbs and optimizes the function objective to protect the analysis results rather than directly perturb the regression results. However, the key issue is how to protect differentiated private information. According to the Stone-Weierstrass Theorem [47], we use the polynomial of *F* as follows.

(21) $$\begin{array}{c} \psi_{i}=\left\{W_{1}^{S_{1}}W_{2}^{S_{2}}\cdots W_{n}^{S_{n}}\;|\;\overset{n}{\underset{m}{\sum }}S_{m}=i\right\}, \end{array}$$

where *ψ*_*i*_ denotes the set of all products of *W*_1_, ⋯, *W*_*n*_. Therefore, the optimization function *F* is formula (22).

(22) $$\begin{array}{c} \overset{I}{\underset{i=0}{\sum }}\underset{\varphi \in \psi_{i}}{\sum }\eta_{\varphi_{f_{i}}}\psi \left(W\right), \end{array}$$

where *η*_*φ*_*f*_*i*___ is the coefficient of *ψ*(*W*). The function perturbation mechanism proposed by HS-DP injects noise into this polynomial coefficient and then obtains the model parameter *W* of the optimized function *F*, as shown in algorithm 1.

**Theorem 1 .**

Let *G* and *G'* be any two adjacent datasets of genes (Assume *G* and *G'* differ in the last tuple), and *F* and *F'* be the objective functions of regression analysis of *G* and *G*′. The polynomial are *F* = ∑_*i*=1_^*I*^∑_*φ*∈*ψ*_*i*__∑_*G*_*i*∈*G*__*η*_*φ*_*f*_*i*___*ψ*(*W*) and *F*′ = ∑_*i*=1_^*I*^∑_*φ*∈*ψ*_*i*__∑_*G*_*i*∈*G*′__*η*_*φ*_*f*′_*i*___*ψ*(*W*), respectively. Then, the inequality is $\sum_{i=1}^{I}\sum_{\varphi \in \psi_{i}}{\left\|\sum_{G_{i\in D}}\eta_{\varphi_{f_{i}}}-\sum_{G\prime_{i\in D}}\eta_{\varphi_{f_{\prime i}}}\right\|}_{1}\leq 2\underset{f}{\max}\sum_{i=1}^{I}\sum_{\varphi \in \psi_{i}}{\left\|\eta_{\varphi_{f_{i}}}\right\|}_{1}$.

**Proof**

Algorithm 1 satisfies *ϵ*-differential privacy.

(23) $$\begin{array}{c} \frac{\text{pr}\left[F\;|\;G\right]}{\text{pr}\left[F\;|\;G^{\prime }\right]}=\frac{\prod_{i=1}^{I}\prod_{\varphi \in \psi_{i}}\exp\left(\varepsilon \cdot {\left\|\sum_{f_{i\in G}}\eta_{\varphi_{f_{i}}}-\eta_{\varphi }\right\|}_{1}/\Delta \right.}{\prod_{i=1}^{I}\prod_{\varphi \in \psi_{i}}\exp\left(\varepsilon \cdot {\left\|\sum_{{f^{\prime }}_{i\in G^{\prime }}}\eta_{\varphi_{{f^{\prime }}_{i}}}-\eta_{\varphi }\right\|}_{1}/\Delta \right.}\leq \overset{I}{\underset{i=1}{\prod }}\underset{\varphi \in \psi_{i}}{\prod }\exp\left(\frac{\varepsilon }{\Delta }\cdot {\left\|\underset{f_{i}\in G}{\sum }\eta_{\varphi_{f_{i}}}-\underset{f_{i}^{\prime }\in G^{\prime }}{\sum }\eta_{\varphi_{f_{i}^{\prime }}}\right\|}_{1}\right),=\overset{I}{\underset{i=1}{\prod }}\underset{\varphi \in \psi_{i}}{\prod }\exp\left(\frac{\varepsilon }{\Delta }\cdot {\left\|\eta_{\varphi_{G_{n}}}-\eta_{\varphi_{G_{n}^{\prime }}}\right\|}_{1}\right),=\exp\left(\frac{\varepsilon }{\Delta }\cdot \overset{I}{\underset{i=1}{\prod }}\underset{\varphi \in \psi_{i}}{\prod }{\left\|\eta_{\varphi_{G_{n}}}-\eta_{\varphi_{G_{n}^{\prime }}}\right\|}_{1}\right),\leq \exp\left(\frac{\varepsilon }{\Delta }\cdot 2\max_{G}\overset{I}{\underset{i=1}{\sum }}\underset{\varphi \in \psi_{i}}{\sum }{\left\|\eta_{\varphi f_{i}}\right\|}_{1}\right),=\exp\left(\varepsilon \right). \end{array}$$

### 4.4. G-Test

At the above stage, multiorder gene combinations strongly associated with disease or phenotypic status have been screened out. At this stage, G-test statistical method [48] was used to verify the significance level of candidate high-order gene combinations.

G-test is a logarithmic likelihood ratio test, and *X*^2^ test is the approximation of the second order Taylor expansion of logarithmic likelihood ratio test. It can be understood that the G-test is more accurate than the *X*^2^ test in some scenarios. Logarithmic likelihood ratio statistics are difficult to calculate, so the *X*^2^ test is widely used. But the G-test is now more widely used when computational power is sufficient. In this paper, we redefined the calculation process of G-test for gene interaction in GWAS, as follows:

(24) $$\begin{array}{c} G=2\overset{I}{\underset{i=1}{\sum }}\overset{J}{\underset{j=1}{\sum }}O_{ij}P_{ij}, \end{array}$$

(25) $$\begin{array}{c} P_{ij}=\left\{\begin{array}{c} a\ln\frac{O_{ij}}{e_{ij}},\overset{J}{\underset{j=1}{\sum }}O_{ij}>\zeta \\ 0,otherwise \end{array}\right., \end{array}$$

where *O*_*ij*_ is the observed number of genotype *I* when the disease state is *y*_*j*_, *e*_*ij*_ is the corresponding expected number of genotype *I* when the disease state is *y*_*j*_, which can be calculated according to the Hardy-Weinberg principle [49].

## 5. Experimental Analysis

In order to protect the privacy of high-order gene interactions and improve the power, a HS-DP framework was proposed in this paper. The above sections have described the main content of HS-DP. In this section, we will verify the performance of this framework through comparing the different algorithms by virtual simulation experiment, still including the source of dataset and the experimental operating environment.

### 5.1. Experimental Setup

There are two types of datasets, simulated and real, of which the simulated dataset was generated by the GAMETES 2.0 software [50]. The sample size of case and control was 4000, respectively, and the SNP number changed within 5000. There were 8 disease models in total, among which models 1-4 were marginal effect models (reference literature [51]. Models 5-8 are generated from the penetrance table with no marginal effect. In addition, we selected age-related macular degeneration (AMD) [52] datasets to judge the practical performance of HS-DP. The framework was trained in a 64-bit Intel(R) Xeon(R) Silver 4210R CPU @ 2.40GHz processor and 32GB RAM simulation environment. And, we used Python 3.6 as the primary programming language in Windows 10. In addition, since the interaction results of the simulated dataset are the last three SNPs, in order to ensure the actual effect of the framework, we distorted this order. Taking the identification of third-order gene interactions as an example, we selected DualWMDR [53] and EDCF [54] algorithms as the comparison algorithms to test the performance of HS-DP proposed in this paper.

### 5.2. Accuracy Comparison Based on Simulated Datasets

#### 5.2.1. With Marginal Effect

**Figure 3:**
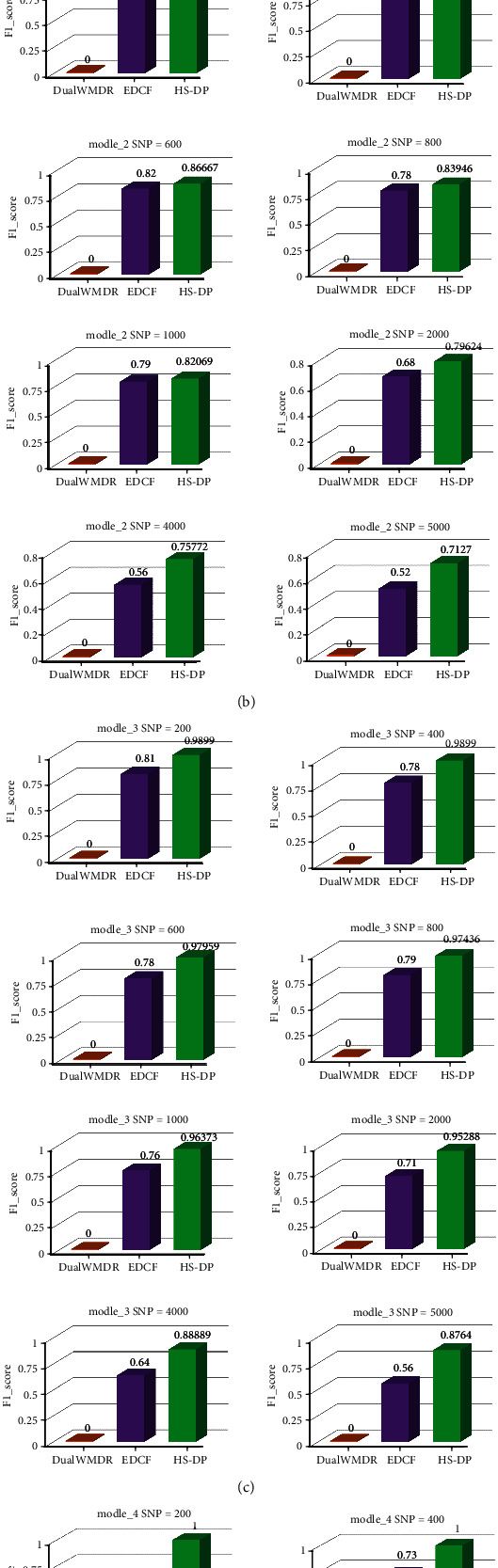
The accuracy of with-marginal effect models.

For models 1 to 4 with marginal effects, we conducted experiments on 9 types of SNP datasets of different sizes. The experimental results are shown in Figure 3.

As can be seen from Figure 3, DualWMDR can identify gene combinations when the number of SNPS in model 1 is 400, 600, and 1000, and model 4 is 400, 600, 800, 1000, and 2000. In other cases, all of the accuracies are 0. It can be concluded that DualWMDR cannot identify most disease models with marginal effects. Although EDCF detects the epistatic combination of genes in most models with higher accuracy than DualWMDR, the accuracy of the algorithm gradually decreases with the increase of data size. The HS-DP algorithm proposed in this paper identifies epistatic gene interactions in 4 disease models and 9 datasets, and the accuracy of the algorithm does not significantly decrease with the increase of SNP size. This is because the harmony search algorithm introduced by HS-DP enhances the search ability of HS-DP for high-order combinations, and five fitness functions integrated that vary on different gradients, which can retain the candidate solution set to the maximum.

#### 5.2.2. Without Marginal Effect

**Figure 4:**
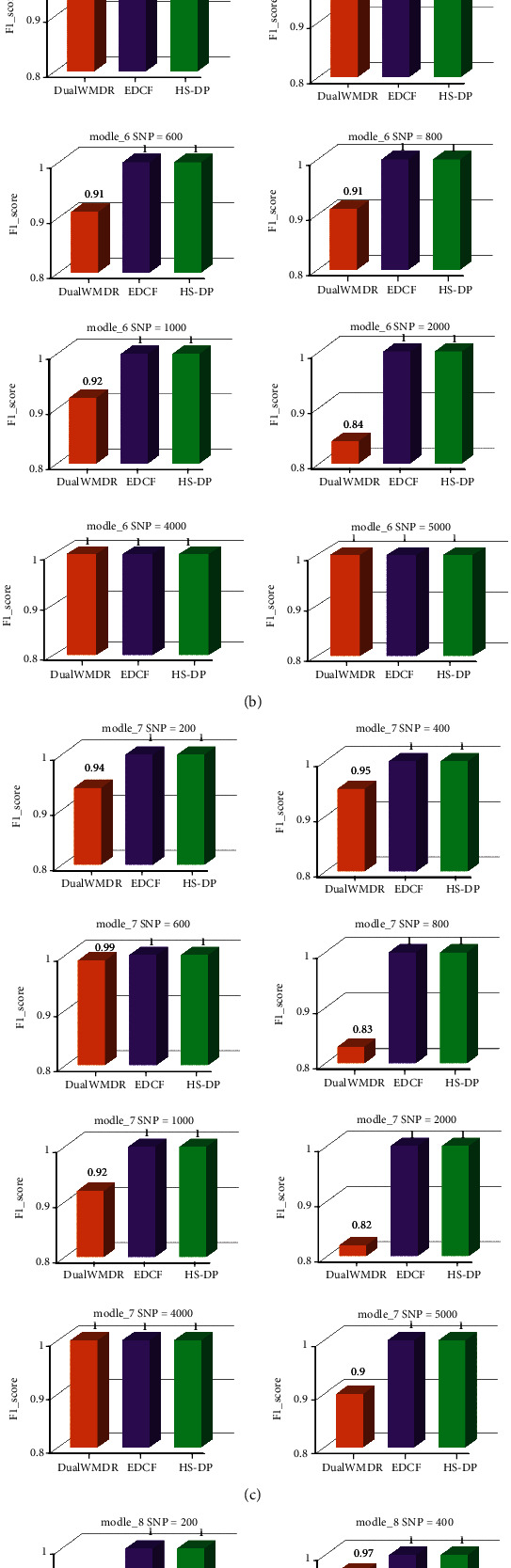
The accuracy of without-marginal effect models.

For models 5 to 8 without marginal effect, we conducted experiments on 9 datasets of different sizes. The experimental results are shown in Figure 4. From Figure 4, experimental results concluded that DualWMDR can find the epistatic genes of without-marginal effect models and 9 kinds of datasets. And the accuracy of DualWMDR does not decrease with the amount of data, it showed that this algorithm is available and has a certain ability to deal with large-scale data. Although EDCF algorithm has high accuracy, it ignores the privacy protection in the research of gene interaction and still has the problem of privacy disclosure. The HS-DP algorithm introduces a differential privacy protection mechanism to solve this problem, meanwhile the detection accuracy is still as high as over 99%.

Based on Figures 3 and 4, it can be concluded that the HS-DP algorithm proposed in this paper not only meets the accuracy requirements of multiple disease models and datasets of different sizes but also protects the privacy and security.

### 5.3. Real Datasets

We tested the accuracy of HS-DP by being applied on age-related macular degeneration (AMD) real datasets. AMD is the leading cause of blindness in middle-aged and elderly people and is a common eye disease. We downloaded AMD data from the official website of WTCCC, which contained 96 case individuals and 50 control individuals with 103611 SNPS. Through quality control, the number of SNP is 96607. Klein et. al [55] reported two interaction results most relevant to AMD, rs380390 and rs1329428. After the initialization parameters, the HS-DP framework took these two results as the main effect SNPS to search for the corresponding third-order gene interaction results in AMD. The results are shown in Table 1.

These are the results of three-order gene interactions on AMD datasets. These SNPs are located in a number of important genes and perform important functions. For example, the CFH gene on chromosome 1 encodes a protein that plays a key role in regulating complement activation. The PCDH9 gene encodes cadherin-associated neuronal receptors and we hypothesized that it is involved in specific neuronal connections and signal transduction. In addition, other combinations of SNPS associated with AMD have been found, but their biological explanation requires further research.

## 6. Conclusion and the Future Work

In order to solve the problem of privacy leakage, improve detection performance, and reduce the detection burden of high-order gene interaction, a secure high-order gene interaction detection framework is proposed in this paper. The framework designed objective function perturbation mechanisms for intelligent algorithms to identify high-order gene interaction combinations. This mechanism added noise according to the distribution characteristics of polynomial data of objective function. In addition, we optimized the process of detecting epistasis by swarm intelligence algorithm and proposed a harmony search algorithm suitable for identification of high-order gene interactions. Experimental evaluations built on simulated and real datasets confirm the accuracy of our framework. In the future, our work will be expanded in the following areas. On the one hand, training convergence is accelerated to improve model accuracy. On the other hand, other noise mechanisms based on differential privacy need to be studied to protect the security of sensitive information from multiple perspectives. Finally, the study of HS-DP on large-scale datasets is also our future research direction.

## Figures and Tables

**Algorithm 1:**
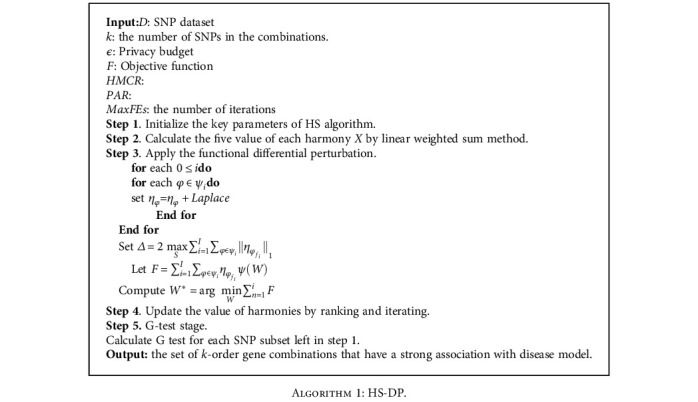
HS-DP.

**Table 1:** Three-order epistatic result of AMD.

Gene	SNP	Location	*P* value
CFH, NPAT, PCDH9	rs380390, rs3781868, rs1036995	11q22, 13q21	8 × 10^−18^
NRG3	rs1458402, rs2207768, rs4901408	11p15	8 × 10^−18^
NXPH1, PTPRD	rs1476623, rs6967345, rs1408120	7p22, 9p23-p24	3.2 × 10^−24^
KANK1	rs595113, rs1569651, rs2031175	9p24	4.9 × 10^−24^
CFH, NPAT	rs132948, rs3781868, rs3781868	1p32, 11p22-23	6.78 × 10^−10^
NAMPT, KCNH7	rs10487833, rs10495593, rs1740752	10p13	3.24 × 10^−18^

## Data Availability

Experimental data is divided into two types, one is simulated data, one is real data (download address: http://www.ncbi.nlm.nih.gov/SNP/).
